# BK Virus-Associated Nephropathy after Renal Transplantation

**DOI:** 10.3390/pathogens10020150

**Published:** 2021-02-02

**Authors:** Yasuhito Funahashi

**Affiliations:** Departments of Urology, Nagoya University Graduate School of Medicine, Aichi 466-8550, Japan; yfunahashi@med.nagoya-u.ac.jp; Tel.: +81-52-744-2985; Fax: +81-52-744-2319

**Keywords:** BK virus, BK virus-associated nephropathy, renal transplantation

## Abstract

Recent advances in immunosuppressive therapy have reduced the incidence of acute rejection and improved renal transplantation outcomes. Meanwhile, nephropathy caused by BK virus has become an important cause of acute or chronic graft dysfunction. The usual progression of infection begins with BK viruria and progresses to BK viremia, leading to BK virus associated nephropathy. To detect early signs of BK virus proliferation before the development of nephropathy, several screening tests are used including urinary cytology and urinary and plasma PCR. A definitive diagnosis of BK virus associated nephropathy can be achieved only histologically, typically by detecting tubulointerstitial inflammation associated with basophilic intranuclear inclusions in tubular and/or Bowman’s epithelial cells, in addition to immunostaining with anti-Simian virus 40 large T-antigen. Several pathological classifications have been proposed to categorize the severity of the disease to allow treatment strategies to be determined and treatment success to be predicted. Since no specific drugs that directly suppress the proliferation of BKV are available, the main therapeutic approach is the reduction of immunosuppressive drugs. The diagnosis of subsequent acute rejection, the definition of remission, the protocol of resuming immunosuppression, and long-term follow-up remain controversial.

## 1. Introduction

The survival of transplanted kidney grafts has improved with the development of novel immunosuppressive therapies. However, among the several types of polyomavirus infectious in humans, since Purighalla et al. first reported in 1995 [1], BK virus (BKV) has been increasingly recognized as the major pathogenic virus after renal transplantation, and it can lead to BKV associated nephropathy (BKVAN). The double-stranded DNA genome of BKV consists of three functional regions: the early viral gene region encoding the regulatory large T- and small t-antigen; the late viral gene region encoding the viral capsid proteins Vp1, Vp2, Vp3, the non-structural agnoprotein, and a pre-microRNA giving rise to miRNA-5p and -3p; and the non-coding control region containing the origin of viral genome replication *ori* as well as regulatory sequences [2,3]. Most individuals become seropositive by approximately four years of age and 70–90% of adults have antibodies to BKV [4,5]. Following primary infection via the respiratory route, BKV remains latent in the renourinary tract, B cells, brain, and spleen [6].

Under the strong immunosuppression after renal transplantation, latent polyomavirus reactivates in the kidney, causing lytic destruction of renal tubular epithelial cells, and resulting in tubular fluid accumulation in the interstitial compartment, which is characterized by an inflammatory interstitial nephropathy, associated with functional impairment due to tubular fibrosis and atrophy [7]. Recently, regular screening for BKV replication by quantitative PCR and pre-emptive reduction of immunosuppression in viremic patients have become common among kidney transplant centers, resulting in a decreased prevalence of biopsy-proven BKVAN [8]. However, a substantial proportion of kidney transplant recipients experience clinically significant BKVAN; viruria in 30–60%, viremia in 10–30%, and BKVAN in up to 10% [6,9,10,11,12,13,14,15,16]. For the treatment of BKVAN, clinicians mainly reduce immunosuppressive drugs, since BKV-specific anti-viral therapy is not yet available. However, a reduction in immunosuppressive drugs can lead to immunological rejection and these treatment failures can lead to transplanted kidney loss. This review summarizes the recent management of BKVAN after renal transplantation.

## 2. Risk Factors

Many clinical studies have reported risk factors for BKVAN. Representative risk factors are renal transplantation from deceased donors, episodes of acute rejection, and Tacrolimus and/or MMF-based maintenance immunosuppression [17,18]. In addition, several factors associated with donors, recipients, and others have been proposed as promoting a high risk of BKVAN development (Table 1). On the other hand, the combined immunosuppression with mTOR inhibitor is reported to promote favorable outcomes [19,20].

## 3. Screening

An allograft needle biopsy is required for a definitive diagnosis of BKVAN [41], but cannot be performed easily due to its invasiveness. Several screening methods have been developed to identify viral activation and renal tissue damage before the development of BKVAN. Such screening permits early diagnosis and reduction in immunosuppressive drugs before the development of irreversible functional impairment of the transplanted kidney. Current guidelines recommend that BKV reactivation should be monitored by regular screening for cytology or viral DNA in urine or plasma [14].

### 3.1. Plasma PCR

Many studies have reported that regular screening for plasma viral load by real-time PCR is useful to identify early viral replication, permit intervention, and prevent progression to BKVAN or allograft loss [10,13,20,42]. Screening for BK viremia can identify at least 90% of patients at risk of BKVAN before significant functional impairment of the renal allograft occurs [43]. Although the optimal frequency and screening methodology remain unclear, the American Society of Transplantation (AST) Infectious Diseases Guidelines recommended regular plasma BKV monitoring of monthly until month 9, and then every three months until two years, then reducing annually until five years [14]. Frequent BKV surveillance should be considered beyond 2 years post-transplantion in pediatric patients because of their high prevalence of BKVAN [44].

Many studies have examined the relationship between urine or plasma BKV levels and diagnosis of BKVAN [6,45,46]. Viscount et al. reported that a plasma BKV DNA level of >1.6 × 4 log_10_ c/mL and a urine BKV DNA level of >2.5 × 7 log_10_ c/mL were strongly associated with BKVAN [46]. Hirsch et al. reported that a plasma BKV DNA level of >4 log_10_ c/mL is recommended for a presumed diagnosis of BKVAN, and the urine viral load in affected patients exceeded 7 log_10_ c/mL [6]. The Kidney Disease: Improving Global Outcomes (KDIGO) clinical practice guidelines also suggest reduction of immunosuppression when the BKV load in plasma is persistently >4 log_10_ c/mL, to prevent disease progression to an irreversible phase [47]. These studies concluded that a presumed diagnosis of BKVAN can be made based on surrogate markers of plasma viral replication. Current guideline mentions that significant BKV replication is assumed when plasma BKV loads of >3 log_10_ c/mL are found in two measurements within three weeks (probable BKVAN), or if loads increased to >4 log_10_ c/mL in at least one of two measurements (presumptive BKVAN) [18]. In these cases with normal baseline renal function, clinicians should start reducing immunosuppression without need for graft biopsy.

In clinical practice, a survey of screening methods among 42 medical centers in the United States reported 42% screening of blood alone, 37% of urine and blood, 17% of urine alone, and 2% of urine cytology [48]. A survey of 90 pediatric physicians in 27 European countries reported that 37% screened blood alone, 37% screened urine and blood, and 26% screened urine alone [49].

### 3.2. Urine PCR

Approximately half of those with high-level viruria develop BK viremia 2–6 weeks later, and again approximately half of the latter are diagnosed with biopsy-proven BKVAN after another 2–6 weeks [50]. Therefore, early signs of viral proliferation in the kidney can be evaluated by quantifying the BKV load in urine. Determining the viral load using PCR addresses weakness in urinary cytology; namely, the lack of specificity and sensitivity, and allows quantification of disease severity [46]. Monitoring BKV in the urine for an early diagnosis of polyomavirus reactivation is valuable for identifying patients at increased risk for BKVAN [51].

The detection rate of BKV in urine varies among studies [9,10,11,12]. Hsieh et al. investigated viral DNA in urine among 250 renal transplant recipients and reported a prevalence of 20.4% [10]. Saundh et al. examined urine in 30 renal-transplant patients at five days and at 1, 3, 6, and 12 months. They reported that 27% were positive for BKV, and BK viruria was mainly detected at 3–6 months after renal transplantation [12]. We also reported that detection of BKV in urine was high during the first six months, and decreased gradually after renal transplantation [11].

Patients with active BKVAN showed a BKV DNA load of >7–8 log_10_ copies/mL in urine samples [6,46,52,53]. However, BKV is latently present in urothelial cells, even in normal populations; therefore, detection of BKV DNA in urine does not always support the presence of a tubulointerstitial disorder. It is generally thought that BK viruria alone, especially in cases with a low urinary BKV load, is of little clinical significance. Screening of healthy adults reported a positive rate of asymptomatic BK viruria of 7% [54]. BK viruria is observed in 8–16% of donors and recipients prior to transplantation [55,56,57]. After renal transplantion, detection of urinary BKV DNA of <5 log_10_ copies/mL without any clinical problems is seen in approximately 10% [54].

Quantification of urine viral load in BKVAN screening is superior to quantification of plasma viral load in several aspects. BKV appears in urine before plasma in the development of BKVAN; negative predictive value of testing urinary BKV DNA is high with a longer period before the onset of viremia and BKVAN. Therefore, quantification of the urine viral load is more suitable to evaluate BKVAN risk at an earlier stage. However, clinicians should interpret a low BKV load in urine cautiously; lower specificity and delayed or lack of viral clearance from the urine after treatment could lead to over-reduction of immunosuppression and the risk of subsequent acute rejection.

Urine PCR test is sensitive but not specific to the diagnosis of BKVAN, therefore, it may be useful for identifying patients at risk before they develop viremia or BKVAN.

### 3.3. Urine Cytology

For the detection of early sign of BKV replication in the transplanted kidney, urine cytology is useful for screening high-risk patients [58]. Virus-infected urothelial cells called “decoy cells”, identified by their typical ground glass intranuclear inclusions on cellular smears stained by the Papanicolaou method, are observed in most patients with BKVAN. Most decoy cells are thought to originate from the renal tubules [59,60,61]. Ariyasu et al. simultaneously immunostained urine specimens with antibodies for S100P and Simian virus 40 (SV40) large T-antigen to determine the origin of decoy cells; 97% of BKV-infected cells originated in the renal tubule [61]. Decoy cell shedding as well as viruria precedes viremia or the development of BKVAN [25]. It can be observed when urine BKV increases to approximately 4–5 log_10_ c/mL [62], when BKV is not amplified in plasma in many cases. However, decoy cells are not specific for the presence of BKV in urine and can be found in JC polyomavirus and adenovirus infection [62,63,64,65].

The correlation between decoy cell number and the severity of renal injury remains controversial. Koh et al. reported that urinary decoy cell number is associated with sustained cell shedding, positive urinary PCR results, and the development of BKVAN [66]. On the other hand, Singh et al. found no correlation between the severity of BKVAN and either decoy cell counts or BKV DNA levels in plasma or urine [67]. Nevertheless, the cytological evaluation of decoy cells in urine is useful in daily practice because of its low cost and non-invasiveness. Many clinicians initially perform urinary cytology tests and then perform PCR if decoy cells are persistently found.

### 3.4. Potential Diagnostic Markers

To overcome the limitations of urinary cytology and urine/plasma PCR for BKV DNA, a lot of biomarkers have been proposed as non-invasive tests for the diagnosis of BKVAN in renal transplant recipients. Singh et al. found that dense, cast-like polyomavirus aggregates termed ‘polyomavirus-Haufen’ were present in fixed voided urine samples by negatively stained electron micrographs and were correlated with the clinical course [68]. In addition, the quantification of its number was correlated with the severity of BKVAN [67]. Mass spectrometric detection of peptides derived from Vp1 has allowed BKV subtypes I and IV to be differentiated [69]. Konietzny et al. reported an association between higher decoy cell numbers and the presence of the Vp1 subtype Ib-2 [70]. In addition, heat shock protein 90-α [71], CXCL9 and 10 [72], Neutrophil gelatinase-associated lipocalin [73], BKV genotyping [74], BKV-specific CD4+ T-cells [75], and urinary exosomal BKV microRNA [76] have been also reported for its usefulness. None of these have been introduced for clinical application.

## 4. Histology

### 4.1. Histological Findings

For diagnosing all of the major allograft disorders, such as acute rejection, chronic allograft nephropathy, and acute tubular necrosis, renal allograft biopsy is the “gold standard”. In BKVAN, a renal biopsy can provide useful information about the extent of renal scarring, type and extent of immune response, and the existence of concurrent pathologies [77].

Since BKV replicates focally in the kidneys, allograft biopsy may sometimes miss the characteristic histological findings of BKVAN, especially when needle biopsies are taken during the early phase of disease or there is a lack of medullary tissue [78]. In BK viremia-positive patients who have multiple biopsy cores collected at the same time, not all biopsy cores show the BKVAN pathology; this is seen in approximately 30% of cases [79]. Therefore, it is recommended that two kidney tissue core samples be collected, at least one of which should contain medullary parenchyma [78]. Allograft biopsy can be skipped in patients with stable renal function because of the possibility of a false-negative result; it should be considered primarily for those with worsening renal function or markers indicating an increased immunological risk, such as a highly sensitized status with panel reactive antibodies, the presence of donor-specific antibodies (DSAs), blood group incompatibility, retransplantation after graft loss due to BKVAN, or a history of acute rejection. When the typical histological findings are not observed despite a high plasma BKV load, a repeat diagnostic biopsy in a different part of the allograft should be considered.

The characteristic histological findings of BKVAN are interstitial inflammation rich in lymphocytes and plasma cells, intranuclear inclusions in tubular epithelial cells, smudgy nuclear chromatin, cellular atypia, and tubular epithelial cell degeneration, along with rounding, detachment, and apoptosis [7,25,80]. Cytopathic changes are more readily observed in the tubules of the medulla. Above all, detecting virus-infected tubular epithelial cells by immunohistochemical staining for SV40 large T-antigen is essential.

Nankivell et al. revealed that the principal driver of the BKVAN phenotype is direct tubular injury caused by the virus, with early acute antiviral responses amplifying the injury, followed by chronic active tubulointerstitial inflammation [80]. Note that it is sometimes difficult to distinguish the histological findings of BKVAN and acute T cell-mediated rejection (TCMR) [81], although they differ in cell type [79,82,83], protein [79], proteomic [84], and gene expression profiles [85,86].

### 4.2. Classification Systems

Several classification systems have been proposed for determining the most appropriate treatment based on the severity of the disease, and for predicting the clinical outcome. Since no single histological finding reliably predicts the clinical outcome, they are combined. The AST and Banff classification systems are widely recognized.

The AST Infectious Disease Community of Practice published a composite system for staging BKVAN based on viral cytopathic changes, interstitial inflammation, tubular atrophy and interstitial fibrosis (Table 2) [14]. The AST classification semi-quantifies the histological findings into patterns A–C, which correspond to acute tubular injury, interstitial nephritis, and severe interstitial fibrosis, respectively. This system especially focuses on the presence of interstitial inflammation and subclassifies pattern B as B1–B3 based on the extent of inflammation. The reported risk of graft loss is <10% in pattern A, 25% in B1, 50% in B2, 75% in B3, and >80% in C [18].

The Banff Working Group retrospectively analyzed patients with “definitive” BKVAN to identify factors associated with the clinical presentation, and found that two independent histological variables were significant: the intra-renal viral load (PyVL score) and the extent of interstitial fibrosis (ci score). They recently updated their classification system (Table 3) and reported graft failure rates of 16%, 31%, and 50% in classes 1–3, respectively [87]. Nickeleit et al. later validated the correlation between the Banff classification and clinical outcome of their recent patients [8]. In higher classes, the time between transplantation and BKVAN diagnosis was longer, the plasma BKV load increased, and peak and long-term serum creatinine levels increased. The graft failure rate was 5%, 30%, and 50% in classes 1–3, respectively.

### 4.3. Immune Response

Inflammatory cell infiltration into the renal allografts was probably the driving force for BKVAN progression. It has been reported that the infiltration of CD3+ (T-lymphocytes), CD4+ (helper T-lymphocytes), CD8+ (cytotoxic T-lymphocytes), CD20+ (B-lymphocytes), CD138+ (plasma cells), and CD68+ (macrophages) cells as well as IL-2R and HLA-DR expression increased with BKVAN progression [88]. T-cells play central roles in the initiation and progression of BKVAN, however, it remains unclear whether BKV responses are mediated predominantly by CD4+ or CD8+ T-lymphocytes, and in particular which subset plays a protective role in the control of the infection [75,89,90]. Large T-antigen preferentially stimulates CD8+ T-cells, whereas Vp1 preferentially stimulates CD4+ T-cells [89]. CD4+ T-cells have a specific polyfunctional antiviral effect on BKV infection by secreting interferon-γ, tumor necrosis factor-α and IL-2 [91]. B-cells and plasma cells also increase with concordant BKVAN progression [79,88], suggesting that humoral immunity is involved in the immunological reaction against BKV.

## 5. Prognostic Factors

In addition to the above-mentioned histological factors included in the classification systems, the risk of graft loss was expected to be higher in cases with high-level viremia, renal dysfunction, deceased donor transplantation, and late acute rejection [79,80,82,92]. Especially, sustained viremia and SV40 large T-antigen positivity on the follow-up biopsy were frequently associated with allograft loss [93,94,95]. Meanwhile, an increase in BKV-specific T cells directed to BKV proteins (large T- and small t-antigen, and Vp1–3) was shown to predict successful control of BK viremia [93,96,97]. Recently, it was found that CD4+ T-cell exhaustion and the diversity of the antigen specific T-cell receptor repertoire affect the BKV clearance time [98].

## 6. Treatment

No specific anti-viral therapy has been developed to treat BKVAN. Thus, early treatment before the development of irreversible histopathological damage are important to ensure a favorable prognosis [25]. The AST guideline recommends starting interventions at the high-level BK viremia stage, which is indicative of probable or presumptive BKVAN [18].

### 6.1. Therapeutic Options

Since no BKV-specific antiviral therapy has been developed, reducing the level of maintenance immunosuppression is the most common and effective treatment for BK viremia or BKVAN. Currently, the AST guideline recommends two strategies [18]: (i) first reduce the dose of the calcineurin inhibitor by 25–50% in one or two steps, and then reduce by 50% and ultimately discontinue the antimetabolites; (ii) first reduce the antimetabolites by 50%, and then reduce the calcineurin inhibitors by 25–50% and discontinue the antimetabolites. At this time, the “calcineurin inhibitor first” and “antimetabolite first” approaches (as the first step) are considered largely equivalent [6,41,43,99,100]. There is in vitro evidence that tacrolimus inhibits anti-BK-specific T cells, which are necessary for viral clearance. Bischof et al. found that reducing the calcineurin inhibitor first in viremia patients led to similar long-term outcomes and clinical rejection rates to those of patients without viremia [99]. They succeeded in clearing the virus from the plasma by reducing tacrolimus in 39% of cases, in another 43% by also reducing mycophenolate, and in 3% by discontinuing mycophenolate (total = 96%). Some centers reduce the antimetabolite first, whereas others discontinue it [6,41]. Hardinger et al. discontinued the antimetabolite first and found that BKV disappeared from the plasma in 95% of viremia patients; half of the patients discontinued the antimetabolite and the other half with sustained viremia also reduced the calcineurin inhibitor [100]. Alternatively, simultaneous dose reduction of both the calcineurin inhibitor and antimetabolite can be considered in severe BKVAN.

Commonly targeted trough levels are tacrolimus < 6 ng/mL, cyclosporine < 150 ng/mL, and sirolimus < 6 ng/mL. Further stepwise reductions may be appropriate in select patients, including those with more advanced disease; in such cases, trough levels of tacrolimus < 3 ng/mL and cyclosporine < 100 ng/mL are targeted [18]. To determine how much immunosuppression reduction is required, clinicians must consider the patient’s immunologic risk, the viral load, and the degree of kidney impairment.

Other options are switching tacrolimus to cyclosporine, switching the calcineurin inhibitor to sirolimus or everolimus, and switching mycophenolate to leflunomide, mizoribine, or everolimus [101,102,103,104].

In addition, several medical treatments have been tried, including intravenous immunoglobulin [105,106], cidofovir [107,108], and fluoroquinolone [109]. Intravenous immunoglobulin through osmotic injury causes vacuolation in proximal tubular epithelial cells, in turn leading to acute kidney injury [110]. Cidofovir is nephrotoxic and has less potent anti-BKV activity than the other therapies [108]. Fluoroquinolone has gastrointestinal and central nervous system side effects [111]. None of these treatments have sufficient clinical data supporting their use as standard practices.

BKVAN sometimes accompanies acute rejection; these cases are difficult to treat. In cases of sustained BK viremia with biopsy-proven acute rejection (with or without evidence of concurrent BKVAN), antirejection treatment should be given first, and immunosuppression reduction should be considered as a second step (e.g., after two weeks). Acute rejection should be diagnosed according to the Banff criteria, while recognizing that tubulitis and peritubular inflammation may also be observed in BKVAN [112,113].

### 6.2. Treatment Goal

During the first two months after diagnosis and reduction of immunosuppression, the serum creatinine levels increase temporally, and tubulointerstitial inflammation worsens in re-biopsy tissues [94,95]. Such increased inflammation (immune reconstitution) clears BKV from the allograft kidney [87,94,95].

The definition of ‘resolution’ is controversial. Some studies define it as the disappearance of SV40 large T-antigen positive cells on a follow-up biopsy and/or negative BK viremia [87,92,95]. However, as the histological changes caused by BKV are seen only in patches of the transplanted kidney, negative viral staining does not always mean the BKVAN has been cured. It is desirable that treatments be continued until there is continuous clearance of plasma BKV DNA. Even then, BK viremia recurs in approximately 10% of patients, who may require an additional reduction of immunosuppression [42,99]. When the immunosuppressant is reduced at the viremia stage, the reported BKV clearance rate is 80–100% [43,99,114], meanwhile, in biopsy–proven BKVAN, the viral clearance rate is lower [114]. In such cases, an additional reduction in immunosuppression over a longer period may be necessary, but treatments sometimes fails resulting in allograft loss.

### 6.3. Treatment-Associated Allograft Rejection

There is concern that reducing immunosuppression can lead to TCMR and antibody-mediated rejection (ABMR). The reported incidence of TCMR is 4.3–14.6% [99,115]. Cheungpasitporn et al. reported that de novo DSA developed in 14% of cases within 14 months of BKVAN diagnosis [116]. These patients had a higher risk of ABMR (hazard ratio 4.75) and allograft loss (hazard ratio 2.63). Other studies demonstrated that BKVAN ultimately resulted in allograft loss in 15–38% of patients; in half of those patients the cause was rejection after immunosuppression reduction rather than uncontrolled viral infection [7,41,80,115].

If the clinical signs of allograft rejection are recognized, such as an acute or progressive rise in serum creatinine despite declining viremia, an allograft biopsy is necessary to determine whether there is concomitant rejection [115]. In such cases, a carefully managed increase in maintenance immunosuppression should be considered, with frequent monitoring of plasma BKV DNA. Treatment response of acute rejection to steroid administration ranges from 40% to 100% [43,117]. When acute ABMR is present, administration of rituximab and plasmapheresis may have to be considered.

### 6.4. Immunosuppressant Resumption

When viral DNA continuously disappears from the plasma on reducing immunosuppression, a return to the baseline level might be required to avoid chronic allograft rejection. There is no consensus on whether a re-increase to the standard dose can prevent subsequent rejection, nor on what drugs should be used, what trough levels should be targeted, and how long viremia should be absent for. Alquadan et al. reported their immunosuppression resumption protocol, which was used in 36 patients with BK viremia [118]. After four weeks of no viremia, they increased mycophenolate by 500 mg/day every 2two weeks up to the standard dose, and then increased the tacrolimus trough levels to 5–7 ng/mL. If viremia recurred during the increase, immunosuppression was reduced again, and then increased stepwise after two months of negative viremia. Only one patient developed low-level viremia, which ultimately resolved. Long-term immunosuppression management after viremia clearance or resolution of BKVAN is individualized based on the balance between the patient’s immunologic risk and the risk of BKV re-activation.

## 7. Retransplantation

Retransplantation is not contraindicated for those who experienced graft failure due to BKVAN. Dharnidharka et al. reported that 126 of 823 kidney recipients were retransplanted after loss of the prior allograft due to BKVAN [119]. The induction and maintenance immunosuppression regimens at retransplantation were similar to those for the first-time; the one- and three-year graft survival rates in retransplanted recipients were 99% and 94%, respectively. Treatment for BKV was reported in 17.5% of retransplanted patients, while graft loss due to recurrent BKVAN was seen in one patient. Leeaphorn et al. compared the clinical outcome of retransplantation in 341 patients who had first graft failure due to BKVAN and 13,260 patients with graft failure for other reasons [120]. The five-year death-censored graft survival rate for the second renal allograft was 90.6% in the BK group and 83.9% in the non-BK group.

There is a consensus that BK viremia should be cleared before retransplantation to minimize recurrence [6,14,41,119,121]. In retransplanted patients with persistent BK viremia, a significant decline of at least 2 log_10_ c/mL is desirable [122].

Surgical removal of the prior transplanted kidney is not necessary at the time of retransplantation, because it does not prevent recurrent BKV replication or BKVAN. However, since BKV-infected allograft nephrectomy rapidly clears BKV from the plasma (t_1/2_, hours to two days) [123], it is an option in cases of uncontrolled BKV replication [122]. Nephrectomy of native kidneys, which remains a reservoir and source of reinfection, is not generally performed. For retransplanted recipients after loss of the prior allograft due to BKVAN, more intense monitoring for BKV replication than usual would be required.

## 8. Conclusions

BKVAN is a threatening complication of renal transplantation. Since early diagnosis and therapeutic management are important, regular screening is highly recommended. Allograft biopsy is required for definitive diagnosis in cases with renal dysfunction, and histological classification systems are useful to predict the therapeutic outcomes. Due to the lack of effective anti-viral drugs, the main therapeutic strategy is to reduce immunosuppression; at the same time, clinicians must pay attention to the risk of allograft rejection.

## Figures and Tables

**Table 1:** Reported risk factors for BKVAN.

Donor factors	Deceased donor [21]
	BKV viruria [22]
	High BKV antibody titers [23,24]
	Female gender [21]
	Degree of HLA mismatches [21,25]
	Positivity of HLA A9 [26], G 3’UTR-4 [27]
	Negativity of HLA C7 [28,29]
Recipient factors	Older age [21]
	Male gender [21]
	ABO incompatibility [30]
	History of hemodialysis [31]
	Low BKV antibody titers [24]
	African American [32]
	Diabetes [21]
	Positivity of HLA A2 [26], G 3’UTR-4 [27]
	Negativity of HLA C7 [28], B51 [33]
Transplant factors	Acute rejection and antirejection treatment [25,34,35]
	Delayed graft function [36]
	Cold ischemia time [37]
	Steroid exposure [38]
	Tacrolimus levels [38]
	Tacrolimus and/or MMF-based maintenance immunosuppression [21,31,39]
	Ureteric stent replacement [40]

**Table 2:** Histological grading of BKVAN–2013 AST classification.

	Pattern A	Pattern B	Pattern C
Viral cytopathic changes	≤25%	11–>50%	variable
Interstitial inflammation	≤10%	B1; 11–25%	variable
		B2; 26–50%	
		B3; >50%	
Tubular atrophy	≤10%	<50%	>50%
Interstitial fibrosis	≤10%	<50%	>50%

**Table 3:** Histological grading of BKVAN–2018 Banff classification.

Class 1		Class 2		Class 3	
PyVL	ci score	PyVL	ci score	PyVL	ci score
1	0–1	1	2–3	-	-
-	-	2	0–3	-	-
-	-	3	0–1	3	2–3

PyVL was semiquantitatively assigned based on the overall percentage of tubules in the cortex and medulla with morphologic evidence of polyomavirus replication. A tubule with intranuclear viral inclusion bodies (type 1 or 2) and/or a positive immunohistochemistry reaction for SV40 large T-antigen in one or more cells per tubular cross section is considered “a positive tubule.” The overall percentage of positive tubular cross sections is estimated in the entire biopsy sample (all available cores, cortex, and medulla). PyVL score 1: ≤1%; PyVL score 2: >1% and ≤ 10%; PyVL score 3: >10% positive tubules/ducts. ci score 0: ≤5%; ci score 1: >5% and ≤25%; ci score 2: >25% and ≤50%; ci score 3: >50% of interstitial fibrosis in cortical area.

## Abbreviations

BKV	BK virus
BKVAN	BK virus-associated nephropathy
KDIGO	Kidney Disease: Improving Global Outcomes
AST	American Society of Transplantation
TCMR	T cell-mediated rejection
SV40	Simian virus 40
ABMR	Antibody-mediated rejection
DSA	Donor-specific antibody
